# *BAIAP2* Is Related to Emotional Modulation of Human Memory Strength

**DOI:** 10.1371/journal.pone.0083707

**Published:** 2014-01-02

**Authors:** Gediminas Luksys, Sandra Ackermann, David Coynel, Matthias Fastenrath, Leo Gschwind, Angela Heck, Bjoern Rasch, Klara Spalek, Christian Vogler, Andreas Papassotiropoulos, Dominique de Quervain

**Affiliations:** 1 University of Basel, Department of Psychology, Division of Molecular Neuroscience, Basel, Switzerland; 2 University of Basel, Department of Psychology, Division of Cognitive Neuroscience, Basel, Switzerland; 3 University of Zurich, Department of Psychology, Division of Biopsychology, Zurich, Switzerland; 4 University of Basel, Psychiatric University Clinics, Basel, Switzerland; 5 University of Basel, Department Biozentrum, Life Sciences Training Facility, Basel, Switzerland; French National Centre for Scientific Research, France

## Abstract

Memory performance is the result of many distinct mental processes, such as memory encoding, forgetting, and modulation of memory strength by emotional arousal. These processes, which are subserved by partly distinct molecular profiles, are not always amenable to direct observation. Therefore, computational models can be used to make inferences about specific mental processes and to study their genetic underpinnings. Here we combined a computational model-based analysis of memory-related processes with high density genetic information derived from a genome-wide study in healthy young adults. After identifying the best-fitting model for a verbal memory task and estimating the best-fitting individual cognitive parameters, we found a common variant in the gene encoding the brain-specific angiogenesis inhibitor 1-associated protein 2 (*BAIAP2*) that was related to the model parameter reflecting modulation of verbal memory strength by negative valence. We also observed an association between the same genetic variant and a similar emotional modulation phenotype in a different population performing a picture memory task. Furthermore, using functional neuroimaging we found robust genotype-dependent differences in activity of the parahippocampal cortex that were specifically related to successful memory encoding of negative versus neutral information. Finally, we analyzed cortical gene expression data of 193 deceased subjects and detected significant *BAIAP2* genotype-dependent differences in *BAIAP2* mRNA levels. Our findings suggest that model-based dissociation of specific cognitive parameters can improve the understanding of genetic underpinnings of human learning and memory.

## Introduction

Human memory is a polygenic trait, characterized by large inter-individual variability. Studies in twins have estimated that heritable factors account for approximately 50% of this variability [1]. Consequently, behavioral genetics studies have identified and characterized genetic variations associated with human memory performance [2], [3]. These findings have been generated either by candidate-gene studies [4]–[7], which depend on pre-existing information, or by genome-wide association studies (GWAS), which allow to identify novel memory-related genes and molecular pathways [8], [9]. However, memory performance is not a result of a single cognitive process, but rather the outcome of many, such as memory encoding, forgetting, or modulation of memory strength by emotional arousal. Animal studies have indicated that the neurobiological and molecular profiles of these processes are partly overlapping and partly distinct [10], [11]. Recent empirical evidence from twin studies also revealed both overlapping and distinct genetic influences on different memory components [12]. Therefore, by relating genetic variability to specific cognitive processes, rather than to general memory performance, additional information about genetic and biological factors involved in learning and memory can be obtained.

Classical behavioral variables of memory performance usually reflect a combination of cognitive processes, any of which may influence the measured variable, making the specific attribution of effect impossible. For example, in spatial learning tasks, latencies to target platform reflect learning but can also be influenced by exploration [13]; in declarative memory tasks the number of recalled items reflects memory, but it also depends on response strategies for weakly remembered items (such as guessing). For this reason, alternative methods, such as computational modeling, can be employed to make inferences about distinct cognitive processes [14] and to study their genetic underpinnings. A number of model-based analysis studies provided useful insights into neural coding of learning rates [15], future discounting [16], exploratory behavior [17], and decision-making under time pressure [18]. Candidate-gene studies related genetic polymorphisms in dopaminergic genes to specific reinforcement learning parameters [19], [20]. Model-based analysis was also used to investigate how stress, motivation, and noradrenergic manipulations influence different reinforcement learning parameters [21], leading to a novel computational interpretation of the inverted-U-shape relationship between stress and behavioral performance. Model-based analyses, however, have not yet been widely used outside the realm of reinforcement learning and decision-making, nor were they applied to GWAS.

In the present study we investigated episodic memory, a memory system that allows conscious recollection of past experiences along with their spatial and temporal contexts [22], [23]. Because aversive emotional arousal is known to strongly enhance memory strength [11], [24], it was the primary focus of our study. We formalized a verbal memory task using a computational model with parameters related to memory encoding, forgetting, emotional modulation of memory strength, and the use of memories in decision-making. Using the best-fitting parameter values for each individual as dependent variables, we performed a GWAS in 1241 healthy young Swiss adults.

## Results

**Figure 1:**
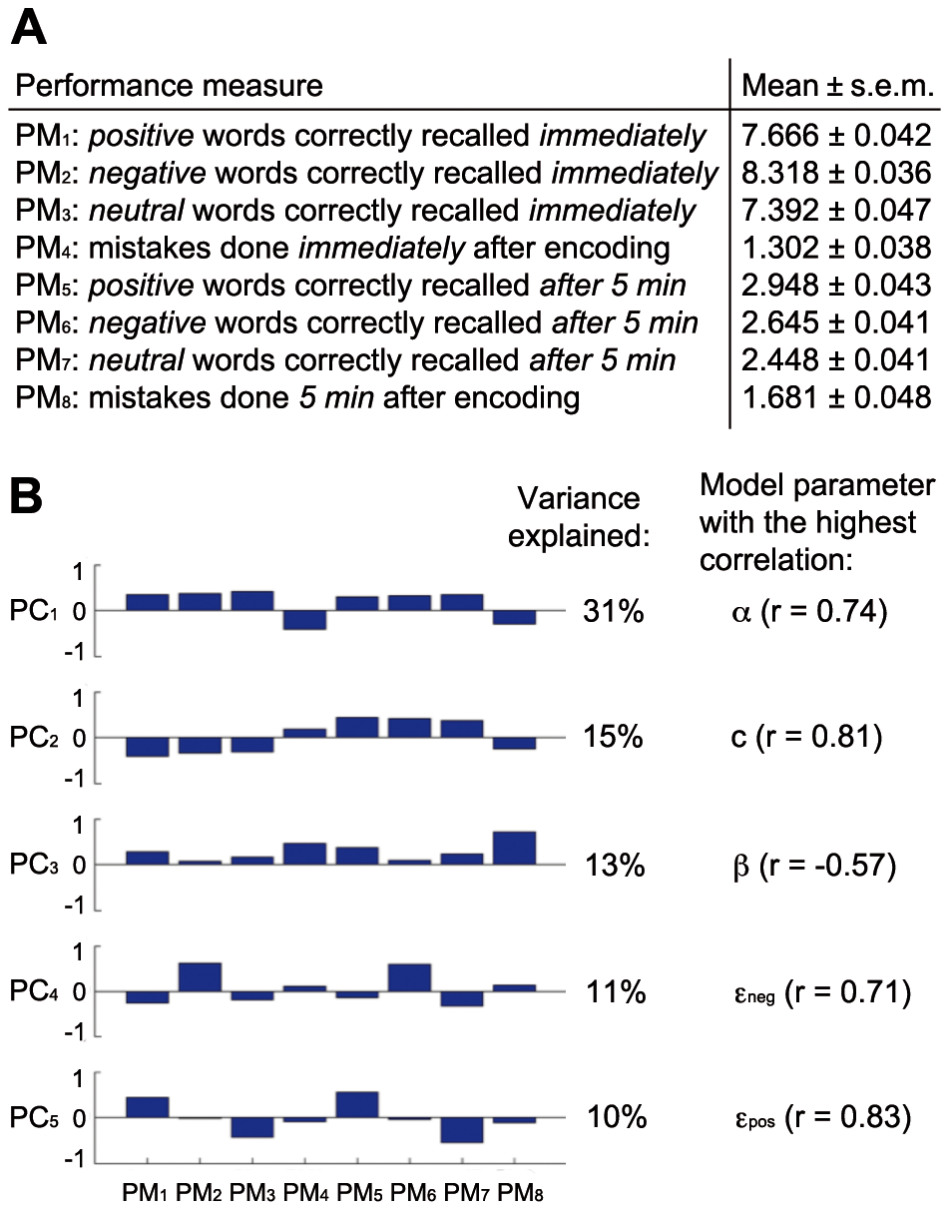
Performance measures and their principal components. (**A**) Description of the performance measures (PM_1−8_) in the verbal memory task and their population statistics. (**B**) Results of principal component analysis: the first five principal components (PC_1−5_) explain 80% of variance in the data; their loadings suggest that the first component (PC_1_) is related to general learning ability, PC_2_ to delayed memory recall (as opposed to immediate recall performance), PC_3_ to mistakes, PC_4_ and PC_5_ to the recall of negative and positive minus neutral words, respectively. Parameters of the best-fitting model that correlate the most with each PC are displayed on the right.

In the verbal memory task we used neutral, positive, and negative words, which had to be recalled at two time points: immediately after the presentation and after a 5 min delay. We characterized behavior using eight different performance measures (PM_1–8_, **Figure 1A**) that indicated the number of correctly recalled words in each valence category as well as the number of mistakes (confabulative errors, i.e. words that were not on the learning list) at the two time points. In line with previous results [7], we observed that most participants recalled emotional words better than neutral ones both immediately and after 5 min (PM_1_>PM_3_, PM_2_>PM_3_, PM_5_>PM_7_, PM_6_>PM_7_, all paired t-test P values <0.0001). The average number of mistakes was higher after 5 minutes compared to immediate recall (PM_8_>PM_4_, P = 2.6 ⋅ 10^−14^) and correlated inversely with the total number of correctly recalled words at both time points (Pearson correlation coefficients r_immed_ = −0.41 and r_5 min_ = −0.26, P values <10^−19^), indicating that participants who have weaker memories are more likely to recall an incorrect (previously unseen) word. To explore essential dimensions of data variability in the population we used principal component analysis (PCA). Applied to the eight performance measures in the verbal task, PCA revealed one component accounting for 31% of the variance, which could be related to general learning ability, and other four components accounting for 10–15% each, which could be related to other aspects of verbal task performance (**Figure 1B**).

### Computational model-based analysis of the verbal task

Although PCA may be the preferred analysis approach in cases where most variance is accounted by few substantial components with insightful and easily interpretable loadings, PCA results usually cannot be directly related to cognitive processes of interest and are strongly dependent on the selection of behavioral variables. Therefore, to dissociate specific cognitive and emotional memory processes, we analyzed performance in the verbal task using a computational model with parameters explicitly related to different cognitive processes. We expected that the model is flexible enough to fit a wide range of individual differences, thereby allowing its best-fitting parameters to be used in GWAS. For each word, the model tracked memory strength *m* that was assigned upon encoding (based on learning rate α and Gaussian noise σ), increased if the word was correctly recalled (based on repetition-based memory improvement *c*), and decreased during the 5 min interval (based on forgetting rate γ). Memory strengths of emotional words were multiplied by positive or negative modulation factors ε_pos_ and ε_neg_ upon encoding. As weak memory traces are not accessible for free recall, we assumed that participants only attempted to recall words with memory strengths higher than decision threshold β. Probability to recall a word correctly was a sigmoidal function of its memory strength (with sigmoidal steepness *s*).

**Figure 2:**
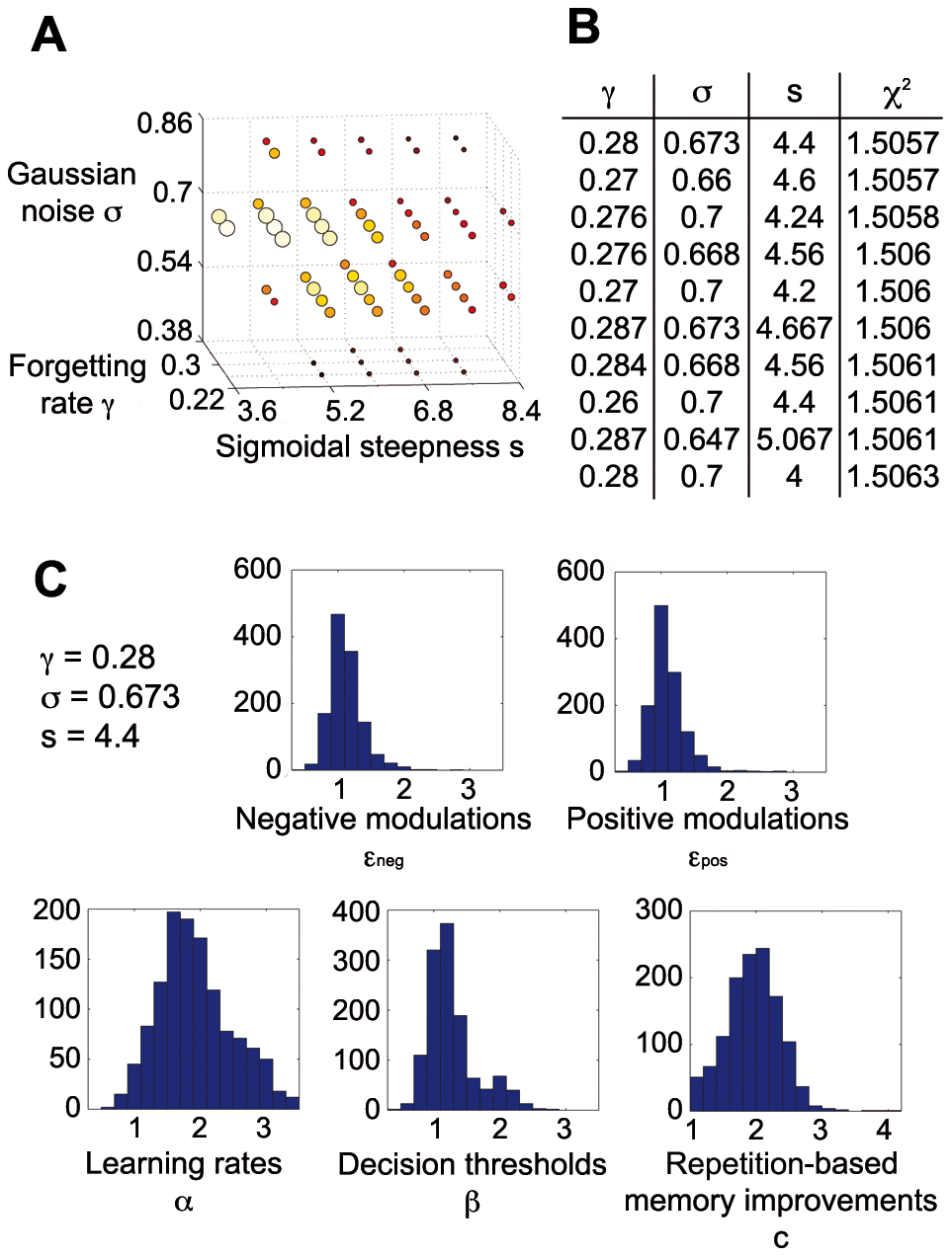
Parameter estimation results for the selected model. (**A**) The hill-climbing results of estimating three fixed parameters (Gaussian noise σ, sigmoidal steepness *s*, and forgetting rate γ) are shown, with bigger circles and lighter colors indicating better goodness-of-fit; ten best hill-climbing points (biggest light yellow circles) were selected for evaluating averages of all their possible combinations, shown in **B**. (**B**) Ten combinations with the best goodness-of-fit (χ^2^) are displayed. Overall, 267 out of 1023 combinations had better χ^2^ than the best hill-climbing point (χ^2^ = 1.522), which suggests that averaging parameters helps overcome step size gaps and leads to refined parameter values. (**C**) Histograms of the best-fitting individual parameters show distributions with the following means: ε_neg_ = 1.12, ε_pos_ = 1.09, α = 1.93, β = 1.27, *c* = 1.95.

As our model had 8 parameters, it was impossible to estimate them for each individual based on only 8 performance measures. Motivated by PCA results that indicated 5 substantial principal components, we chose to estimate 5 parameters individually, with the remaining 3 kept fixed among the population. To avoid selecting the most subjectively interesting parameters, we performed an empirical model selection procedure, evaluating goodness-of-fits of models with different free and fixed parameters and selecting the best-fitting model. Due to computational constraints, this procedure was performed in several stages with different accuracy (see Materials and Methods, **Table S1,** **Figure 2** **, Figure S1**), leading to the selection of learning rate α, decision threshold β, repetition-based memory improvement *c*, positive and negative modulation factors ε_pos_ and ε_neg_ as free parameters, estimated for each individual, whereas Gaussian noise σ, forgetting rate γ, and sigmoidal steepness *s* were estimated for the whole population (**Figure 2**). More than 99% of individually estimated parameter sets passed the χ^2^ test of goodness-of-fit (satisfying P(χ^2^, ν) > 0.05, mean χ^2^ = 1.5057), thus our model was sufficiently flexible to reproduce a wide range of behavioral phenotypes. High correlation coefficients (mean r = 0.95) and low standard deviations (on average 3.4% of the respective range) among the 10 best parameter sets (hill climbing end points) for each individual indicated that estimated parameter values were reliable. Except the lower bound of repetition-based memory improvement *c* = *1* (as repetition should not weaken memories), 99.9% of individually estimated parameter values belonged to the middle 90% of the value ranges, suggesting that the selected parameter estimation bounds did not constrain the results. Moreover, each of the five most significant principal components showed moderate to strong correlation to a different model parameter (**Figure 1B**), suggesting that these five parameters represented the most relevant dimensions of variance in the population.

### Negative modulation of memory is associated with *BAIAP2*

We used individually best-fitting model parameters for GWAS of the verbal task. All DNA samples from participants who underwent the verbal memory task were processed on the Affymetrix® Genome-Wide Human SNP Array 6.0 in one centralized microarray facility. After excluding SNPs that had high missing genotype rate, low minor allele frequency, or deviated significantly from Hardy-Weinberg equilibrium, a total of 587111 out of the 930856 array SNPs were used for association analyses under an additive genetic model. After controlling for population stratification and age effects, 1241 participants entered the final GWAS. As distributions of 4 parameters (α, β, ε_pos_, ε_neg_) were not normal (Lilliefors test P<0.001), we used Spearman rank correlation for evaluating statistical significance of the genetic associations.

The highest level of statistical significance was observed for the association between a marker SNP rs8067235 in the brain-specific angiogenesis inhibitor 1-associated protein 2 gene (*BAIAP2*, HGNC:947) and negative modulation of memory strength ε_neg_. This association survived Bonferroni correction for genome-wide multiple comparisons (P_nominal_ = 5.5 ⋅ 10^−8^, P_Bonferroni_ = 0.032). There were no further Bonferroni-corrected associations. The effect had a similar magnitude in the two GWAS sub-samples: Zurich and Basel (Spearman's ρ_total sample = _0.154, ρ_Zurich = _0.139, ρ_Basel = _0.167, **Table 1**). To take the uncertainty of parameter estimation into account, we performed a bootstrapping procedure where 10000 samples were generated as random combinations of the 10 best-fitting individual parameter sets. Despite additional variability, the association between rs8067235 and ε_neg_ remained highly significant (the median P value among the 10000 samples was P_nominal_ = 6.5 ⋅ 10^−8^, P_Bonferroni_ = 0.038). Nominally significant associations with rs8067235 were also apparent in the analysis of classical performance measures that can be related to negative modulation of memory but are less specific than ε_neg_ (**Table S2**). However, the effect sizes were lower compared to ε_neg_, indicating that analysis of such measures alone would not have led to the discovery of the reported association.

**Table 1:** Association between *BAIAP2* rs8067235 genotype and model parameter ε_neg_ in the verbal memory task.

rs8067235 genotype	Combined sample (N), ε_neg_ mean±s.e.m.	Zurich subsample (N), ε_neg_ mean±s.e.m.	Basel subsample (N), ε_neg_ mean±s.e.m.
*AA*	(137) 1.20±0.02	(64) 1.20±0.03	(73) 1.20±0.03
*AG*	(532) 1.14±0.01	(254) 1.13±0.02	(278) 1.14±0.02
*GG*	(570) 1.08±0.01	(264) 1.09±0.01	(306) 1.08±0.01
	P = 5.5 ⋅ 10^−8^	P = 8.0 ⋅ 10^−4^	P = 1.7 ⋅ 10^−5^
	ρ = 0.154	ρ = 0.139	ρ = 0.167

Significance is calculated based on the additive genetic model. ρ: Spearman's rho

To better characterize the profile of the genetic association signal in the *BAIAP2* locus we used data from the 1000 Genomes project [25] and reinvestigated this region using imputation (**Text S1**), which allowed for analysis of virtually all common SNPs in this region and offered a sevenfold increase in marker density over the 6.0 array SNPs. Imputation analysis confirmed the initially observed pattern of association and revealed highly significant intragenic SNPs and rapid decrease in significance with increasing distance from the genome-wide significant locus (**Figure S2**). Haplotypic structure further around the *BAIAP2* locus indicated no associations with SNPs of the neighboring genes. To prevent false interpretations due to possible array-related genotyping errors, SNP rs8067235 was additionally genotyped on a different, singleplex platform (**Text S1**). The level of convergence between array- and singleplex-based genotype calls was 100%.

In an independent population of 451 healthy young subjects we investigated if the association between *BAIAP2* SNP rs8067235 and the modulation of memory strength of words by negative emotional valence also translated to the amount of remembered negative information as assessed by free recall of pictures. Here the number of correctly recalled pictures in each emotional valence category and the number of mistakes were recorded 10 min after encoding. As in the verbal task, we observed that most participants recalled negative pictures (proportion recalled±s.e.m. = 46.2%±0.6%) better than neutral ones (28.3%±0.6%; paired t-test P = 6.3 ⋅ 10^−103^). In this task, the phenotype that was most related to ε_neg_ was the difference between proportions of correctly recalled negative and neutral pictures. We found that it was significantly associated with rs8067235 in the same direction as the original association discovered in the verbal task GWAS (**Table 2**).

**Table 2:** Association between *BAIAP2* rs8067235 genotype and performance measures related to negative modulation of picture memory.

rs8067235 genotype	Proportions of negative pictures recalled after encoding	Proportions of neutral pictures recalled after encoding	Proportions of negative minus neutral pictures recalled after encoding
*AA* (N = 47)	0.490±0.021	0.293±0.019	0.197±0.019
*AG* (N = 200)	0.475±0.009	0.284±0.009	0.191±0.010
*GG* (N = 204)	0.443±0.009	0.280±0.008	0.163±0.009
	P = 0.003	P = 0.714	P = 0.013
	ρ = 0.141	ρ = 0.017	ρ = 0.117

Significance is calculated based on the additive genetic model. ρ: Spearman's rho. Values denote mean±s.e.m.

.

### *BAIAP2* variants show differences in parahippocampal activity

As *BAIAP2* SNP rs8067235 was associated with modulation of memory strength by negative emotional information in the word and picture tasks, we investigated potential neural correlates of this association using the subsequent memory paradigm [26], [27], applied to the event-related fMRI. In this paradigm the differential activity during encoding of subsequently remembered vs. subsequently forgotten pictures, known as the Dm (difference due memory [26]), is thought to reflect successful encoding processes. The medial temporal lobe (MTL) memory system, consisting of hippocampus, amygdala, parahippocampal and entorhinal cortices, has been consistently implicated in successful encoding as well as memory modulation by emotional information [24], [28]–[30]. For this reason we defined the MTL memory system as our region of interest (ROI).

**Figure 3:**
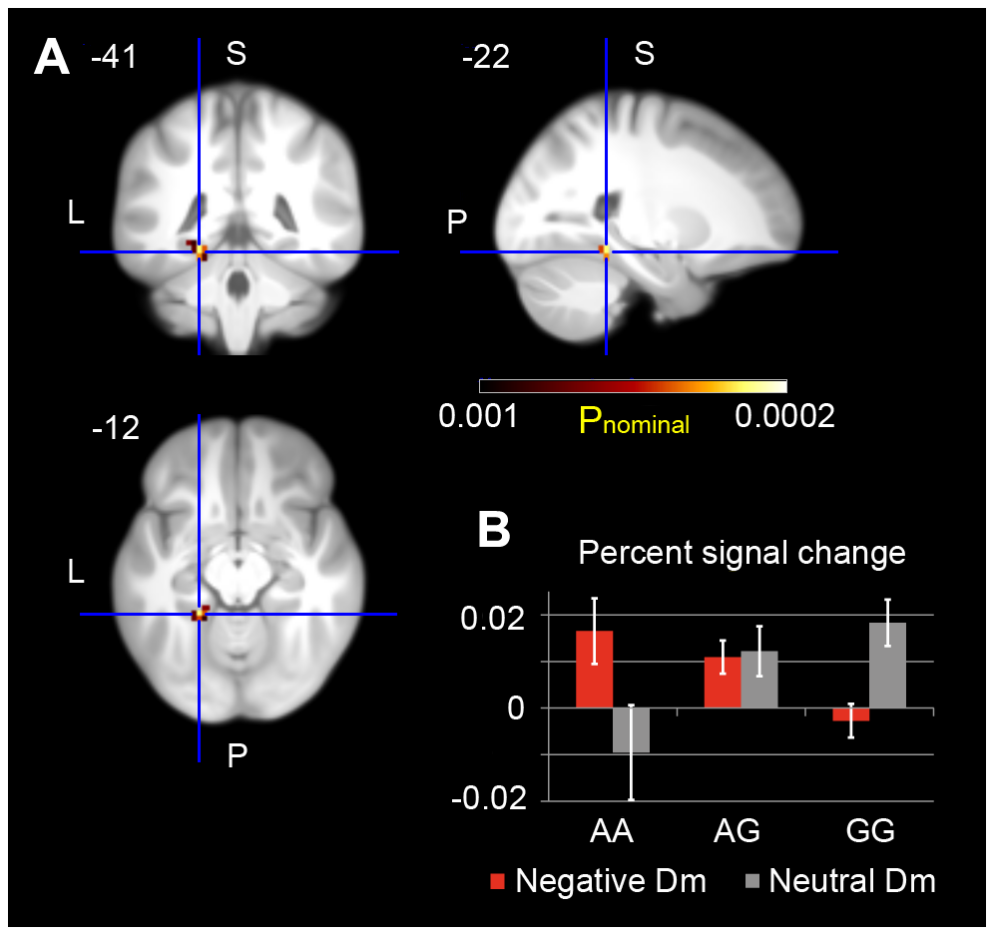
*BAIAP2* rs8067235 genotype-dependent differences in brain activity specifically related to negative modulation of memory strength. (**A**) Displayed are gene dose-dependent (with increasing number of *A* alleles) activity increases in left parahippocampal cortex (peak MNI coordinates [−22 −41 −12], Z(max) = 3.50, *P*_nominal_ = 2.3 ⋅ 10^−4^, *P*_small-volume-FWE-corrected_ = 0.033). Activations are overlaid on coronal (upper left), sagital (upper right), and axial sections of the study specific group template, displayed at an uncorrected threshold of P = 0.001 and using color-coded P values (number of voxels in the cluster: k = 10). L, left side of the brain; P, posterior; S, superior. (**B**) Genotype-dependent dissociation of negative and neutral Dm effects in left parahippocampal cortex (at the peak activation [−22 −41 −12]): progression from AA to GG genotype leads to shift in the parahippocampal sensitivity from negative to neutral Dm.

The fMRI data was available for 435 subjects who performed the picture task. We first investigated which parts of the MTL memory system showed a Dm effect for negative or neutral items. Clusters in amygdala, hippocampus, and to a lesser extent entorhinal and parahippocampal cortices were sensitive to negative Dm, whereas parahippocampal and hippocampal clusters were sensitive to neutral Dm (**Table 3**). These genotype-independent results were consistent with previously reported dissociation between anterior and posterior MTL regions in their sensitivity to emotional vs. neutral subsequent memory [29]. We hypothesized that rs8067235 genotype-dependent differences in negative vs. neutral memory could translate to differences in negative vs. neutral Dm effects in some of these clusters. This analysis revealed gene dose-dependent (with increasing number of *A* alleles) increases in activity in the left parahippocampal cortex (peak activation at [−22 −41 −12]; *P*_small-volume-FWE-corrected = _0.033, **Figure 3A**) that were related to differences between negative and neutral Dm. fMRI signal changes at the peak activation indicated genotype-dependent dissociation of left parahippocampal sensitivity to neutral vs. negative Dm: GG carriers showed a Dm effect for neutral items, AA carriers were sensitive to negative Dm, whereas AG carriers showed sensitivity to both types of Dm, albeit at a smaller magnitude (**Figure 3B**). Differences between individual negative and neutral Dm effects at the peak activation were correlated with the differences between numbers of correctly recalled negative and neutral pictures (r = 0.113, P = 0.009), suggesting as well that independently of genotype, left parahippocampal activation reflects the extent to which negative valence affects memory strength. Outside of our defined ROI, we did not observe any rs8067235 genotype-dependent activation differences that survived correction for multiple comparisons.

**Table 3:** Genotype-independent subsequent memory (Dm) analysis for negative and neutral pictures.

Contrast	Region	No. of voxels	L/R	Peak MNI coordinates (x, y, z)	T value	P value
Negative Dm	amygdala	60	R	19, −6, −16	7.94	<10^−6^
		46	L	−22, −6, −16	7.83	<10^−6^
Negative Dm	hippocampus	84	R	19, −11, −16	6.11	<10^−6^
		71	L	−17, −11, −16	5.06	<10^−6^
Negative Dm	parahippocampal cortex	25	R	19, −28, −16	5.71	<10^−6^
		7	L	−28, −30, −20	3.73	0.0001
Negative Dm	entorhinal cortex	10	L	−30, −8, −32	4.95	<10^−6^
		3	R	30, −2, −36	3.84	0.0001
Neutral Dm	parahippocampal cortex	23	L	−17, −41, −12	4.24	10^−5^
		14	R	25, −41, −8	3.67	0.0001
		2	L	−17, −19, −24	3.37	0.0004
Neutral Dm	hippocampus	9	L	−28, −36, −8	4.08	3 ⋅ 10^−5^
		8	L	−17, −17, −24	3.87	0.0001
		2	L	−11, −39, 4	3.37	0.0004

### Clusters with voxels at P<0.001 significance level are shown.*BAIAP2* variants show differences in mRNA expression

SNP rs8067235 is located within an *H3K27Ac* histone mark and a DNaseI hypersensitivity site [31], both of which are indicative of genomic regions involved in transcriptional regulation and activity (**Figure S3**). To study the possible genetic association between *BAIAP2* and *BAIAP2* mRNA expression levels, we analyzed the cortical expression of the *BAIAP2* transcript GI_9257196 (NM_017450.1) in the brains of 193 non-demented deceased subjects [32]. SNP rs8067235 is not represented on the 500 k SNP Array set, which was used in the study of cortical gene expression. We therefore analyzed SNP rs8070741, which was the closest linked array SNP (r^2^ = 0.34; D′ = 0.816; χ^2^ = 320, df = 4, P<0.0001). The total of 193 individuals were distributed among the three genotypic groups as follows: 63 *GG* carriers, 93 *AG* carriers, and 37 *AA* carriers (P_HWE_ = 0.8). Comparison between genotype groups revealed statistically significant genotype-dependent levels of *BAIAP2* mRNA (**Figure S4**).

## Discussion

By employing a computational model to estimate individual cognitive parameters and using them for GWAS we found an association between a common polymorphism (rs8067235) of *BAIAP2* and negative emotional modulation of memory strength. In addition to the verbal task, where carriers of rs8067235 A alleles – as compared to non-carriers of the A allele – had higher values of negative modulation parameter ε_neg_, in the picture task they also showed better free recall of negative compared to neutral pictures and higher neural activity in left parahippocampal cortex that was specifically related to successful encoding of negative compared to neutral pictures. In addition, we detected *BAIAP2* genotype-dependent differences in *BAIAP2* mRNA levels in the human cortex.

Previous studies found that *BAIAP2* plays a role in neuronal growth cone guidance [33], and its mouse homologue *IRSp53* was implicated in NMDA receptor-mediated excitatory synaptic transmission, long-term potentiation, and spatial learning [34]. Genetic variations in *BAIAP2* were also associated with attention-deficit hyperactivity disorder [35] and autism [36]. A functional neuroimaging study [29] showed that posterior MTL areas were more sensitive to neutral subsequent memory and anterior ones to emotional subsequent memory, which was essentially replicated in our genotype-independent fMRI results (**Table 3**). However, our *BAIAP2* rs8067235 genotype-dependent analysis also revealed that for a relatively small group of individuals (*AA* carriers) their parahippocampal cortex was sensitive to negative, not neutral subsequent memory (**Figure 3B**), suggesting that individual genotype may affect the boundaries or balance of negative and neutral encoding in the MTL memory system.

Aside from biological implications of our results, the use of computational modeling for GWAS of human memory is an important methodological development. Model-based analysis allows incorporating and studying important hidden variables that are not amenable to direct observation [14]. As memory performance is the result of distinct cognitive processes subserved by partly distinct molecular profiles, model-based analyses can dissect a raw behavioral phenotype to specific cognitive and emotional memory parameters. Such approach can address a number of different scientific questions (e.g. genetic associations with immediate, long-term memory, emotional modulation, and decision-making) in a single study, based on a single experiment. In the context of our study it is also important to stress that conventional GWAS, restricted to the directly observable behavioral phenotypes, would have missed the association between *BAIAP2* variants and emotional modulation of memory strength.

For practical reasons (such as limited dimensionality of the data and feasibility of parameter fitting), our computational model contains some simplifications of the modeled cognitive processes. Nevertheless, our model takes into account most of the relevant processes without prior assumptions on which parameters are of interest and which should be fixed. Although estimated parameters may depend on the model design, in some cases critically [37], it is important to consider that *any* model of such kind is a substantial simplification of the underlying neural mechanisms, thus it is unavoidable that some subtle aspects will always be missed. However, the merits of model-based studies should not be judged in isolation, but compared to the alternatives, such as raw behavioral variables or their principal components, which often lack specificity, interpretability and may not generalize to different populations, tasks, and phenotypes. Even very simple models are useful if they are supported by empirical evidence such as neural or genetic correlates, which can enable prediction of individual cognitive parameters based on various modulatory factors (as was shown in the model-based study of mouse behavior [21]). Such predictive capabilities will ultimately help design efficient, simulation-based means to test cognitive and pharmacological manipulations that could be useful for improving cognitive abilities and treating neuropsychiatric disorders.

## Materials and Methods

### Ethics statement

After complete description of the study to the subjects, written informed consent was obtained. The experiments were approved by the ethics committees of the Cantons of Zurich and Basel, Switzerland.

### Participants and data pre-processing

We recruited healthy, young Swiss subjects in 3 samples: the Zurich words sample (192 males, 514 females, age mean±standard deviation = 21.92±2.95 years), the Basel words sample (261 males, 504 females, age 22.47±3.62 years), and the Basel pictures/fMRI sample (207 males, 324 females, age 22.54±3.26 years). A total of 930856 SNPs were genotyped (**Text S1**). For association testing markers with call rate less than 0.95, with minor allele frequency less than 0.05, and with Hardy-Weinberg equilibrium *P*<0.05 were excluded leaving a total of 587111 markers to be analyzed. After outliers were excluded based on population stratification and age (**Text S1**), the following numbers of participants remained for the final analysis: 584 in the Zurich words sample, 657 in the Basel words sample, and 451 in the Basel pictures/fMRI sample.

### Memory testing – the verbal task

Subjects viewed six series of five semantically unrelated nouns presented at a rate of one word per second with the instruction to learn the words for immediate free recall after each series. The words were taken from the collections of Hager and Hasselhorn [38] and consisted of 10 neutral words such as “angle”, 10 positive words such as “happiness” and 10 negative words such as “poverty”. The order of words was pseudorandom, with each group of 5 words containing no more than 3 words per valence category. In addition, subjects underwent an unexpected delayed free-recall test of the learned words after 5 min (episodic memory). The free recall of a word was considered successful only if it was spelled correctly or a with single letter typo that did not make it become a different valid word (multi-letter typos were very rare). The relevant performance measures (PMs) are described in **Figure 1A**.

### Computational model for the verbal task

To dissociate specific cognitive processes involved in learning and memory, we used a computational model to describe individual performance in the verbal memory task. The key assumption of the model is that depending on how well individuals remember a word they may or may not try to write it down in the free recall, and if they try, their recall may or may not be correct. The probability that the attempted recall is correct depends on *memory strength m* of each word (which is the main variable of the model) as follows: 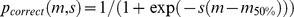, where the sigmoidal curve is described by *steepness s* and center of the sigmoid chosen as m_50%_ = 1 (any positive constant could be used here, the definition would become equivalent if some other parameters are scaled proportionally). The decision of whether to attempt the recall of weak memories depends on one's willingness to risk making errors, which varies between the individuals. We chose to model this decision-making aspect using *decision threshold* β, where words with memory strength m > β were attempted to be recalled, whereas those with m<β were not. As a result, individuals with high β values did not attempt recalling weakly remembered words, leading to fewer recalled words but also avoiding the confabulative errors (i.e. words that were not on the learning list), whereas individuals with low β values did more guessing, leading to a higher number of recalled words but also to more errors.

During encoding, the initial memory strength for each word was assigned as 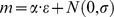, where α was *learning rate*, ε emotional modulation of memory (ε = ε*_neg_ for negative words*, ε = ε*_pos_ for positive words*, and ε = 1 for neutral words), and N(0, σ) the Gaussian noise with mean 0 and *standard deviation* σ, reflecting randomness in learning different words. As the memory strength of words that have been recalled and written down in the immediate recall is likely to increase due to repetition, we multiplied the memory strength m of immediately recalled words by a *repetition-based memory improvement c* (*c*≥*1*). Forgetting during the 5 min delay was formalized by multiplying all memory strengths by *forgetting rate γ* (*γ*<*1*).

Eventually our model had 8 parameters: learning rate α, decision threshold β, forgetting rate γ, positive memory modulation ε_pos_, negative memory modulation ε_neg_, sigmoidal steepness *s*, repetition-based memory improvement *c*, and standard deviation of the noise σ. However, it was impossible to estimate all of these parameters individually for several reasons: first, some of them were closely related to each other, thus keeping such parameters all free would compromise stability and reliability of the estimation; secondly, our behavioral phenotype consisted of only 8 measures per individual, too few to reliably infer 8 parameters. Motivated by the results of principal component analysis (that indicated five substantial and meaningful components, see **Figure 1B**, with the remaining three accounting for only 6–7% of variance each), we chose to set 5 of these parameters free (different between individuals) and 3 remaining ones fixed (same for all individuals). The selection of which parameters would be free and which fixed was done based on the corresponding mean goodness-of-fit values (i.e. empirical selection of the most appropriate model was performed).

### Estimation and evaluation of best-fitting model parameters

For the estimation of best-fitting model parameters we computed expected values of all performance measures (PM_1-8_, see **Figure 1A**) as a function of 8 model parameters (α, β, γ, ε_pos_, ε_neg_, σ, c, s). Computing integrals over probability distributions of memory strength *m* (**Text S1**) was a more efficient and robust approach than simulating the model with random numbers and computing averages over multiple simulation runs. Integrals were computed numerically using Matlab 2008a (The Mathworks Inc., Natick, MA, USA). As a control, we also simulated the model stochastically: averages of PMs over 100000 simulations were almost exactly the same as using the expected-value-based method. To evaluate how well the model with a particular set of parameters fits individual behavioral performance, we used the following goodness-of-fit function [21], [39]: 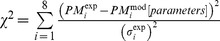 where PM_i_^exp^ and PM_i_^mod^ are experimental and modeled performance measures of that individual, respectively, and (σ_i_^exp^)^2^ is the variance in the experimental data of PM_i_. With χ^2^ as the objective function to minimize, we performed the estimation of best-fitting parameters in several stages:

- “Model selection”: to determine which five parameters should be estimated individually, we evaluated all 56 possible 5-out-of-8 combinations. Because of high computational cost of running 56 full estimation procedures, at this stage we performed only a moderately accurate estimation of the three fixed parameters.
- Using two best models, we performed a more refined estimation of fixed parameters, thereby improving the χ^2^ values. We note that although improvements of χ^2^ values were substantial, they were small compared to the differences between the initial χ^2^ values of the two best models and other worse models; therefore, it is very unlikely that any of those other models would become comparatively better due to refinement.
- For the final refinement, we evaluated the averages of all 2^10^–1 = 1023 combinations of the 10 best parameter sets for each model, thereby further improving the χ^2^ values. Finally, parameter sets from the model with the best goodness-of-fit were used for the GWAS.

In all parameter estimation steps the search was performed in the following ranges: (α, β, ε_pos_, ε_neg_, σ) ∈ [0.3, 3.5], c ∈ [1, 4.2], γ ∈ (0, 0.8], and s ∈ (0, 16]. In choosing the ranges we had to balance two partially opposing aims: keep these ranges as similar as possible to avoid possible bias to estimation results, and keep them as close to a likely distribution of each parameter as possible to maximize estimation accuracy. The most often used range, [0.3, 3.5], was chosen after some preliminary estimation runs, ensuring that less than 1% of estimated parameter values are near the boundaries, but histograms of the estimated parameters cover a substantial part of the range. For other parameters the ranges were modified either due to fundamental constraints (c > 1 and γ<1) or because the likely spread of parameter values would be very different from the default range (for γ and s).

#### Stage 1

To estimate the best-fitting parameters for each individual, we first generated 8^5^ = 32768 sets with each of the 5 free parameters assigned a value at regular intervals (1/16, 3/16, 5/16, 7/16, 9/16, 11/16, 13/16 or 15/16 fraction of its respective range), whereas the 3 fixed parameters were searched among 4^3^ = 64 sets by assigning them a value at 1/8, 3/8, 5/8 or 7/8 of their respective range. Out of these 64 sets, 20 fixed parameter sets with best average goodness-of-fit were chosen for further estimation. For each chosen set of fixed parameters 10 best-fitting parameter sets per individual were used as starting points of the hill climbing procedure, where steps along each parameter (in both directions, step size = 5% of the respective range) were examined until an improvement in the χ^2^ value could be found (and then continued iteratively, until no further improvement was possible). The order of gradient descent steps was determined using pseudorandom numbers (i.e. it remained the same if the same estimation were repeated multiple times), as random noise would make the estimation of fixed parameters unreliable. The average of hill climbing end-points was also evaluated, and if the resulting χ^2^ value was better than of all single end-points, it was used further.

Secondly, keeping estimated individual parameters fixed, we performed a similar hill-climbing procedure for fixed parameters (with step sizes = 5% of the respective range). Finally, with new fixed parameter values we repeated the hill climbing along individual parameters, but now using smaller steps (step size = 1% of the respective range). The resulting goodness-of-fit averages (over all individuals) of models with best-fitting individual and fixed parameters were used to select the 2 best models for further refinement of fixed parameters. Such refinement was necessary because so far we only performed hill climbing along individual parameters with fixed parameters being fixed or vice versa. Performing both hill climbing procedures simultaneously would have been too computationally costly for 56 different models.

#### Stage 2

The refinement of estimated fixed parameters was performed in the following way: starting from the 2 best fitting fixed parameter sets for each model, we performed steps of 5% of the respective range in both directions along each of the three parameters. At each step we performed the same estimation of best-fitting individual parameters as above and *all* steps that resulted in improved average goodness-of-fit over all subjects were used as starting points for further hill climbing.

#### Stage 3

Finally, the 10 best resulting sets of fixed parameters (and the corresponding best-fitting individual parameters) for each model were used to evaluate the goodness-of-fit of all 2^10^ – 1 = 1023 averages of their possible combinations.

To evaluate how well the model fits individual data, we used the χ^2^-test with ν = 8–5 = 3 degrees of freedom (5 free parameters and 8 PMs). For each individual, we calculated the P(χ^2^, ν) value, defined as the probability that a realization of a χ^2^-distributed random variable would exceed χ^2^. Values of P(χ^2^, ν) > 0.05 indicate no statistical difference between modeled and observed PMs, meaning that the model fits the data well. In addition to the χ^2^-test, goodness-of-fit could be evaluated based on correlations across the population between experimental and modeled PMs – high correlations indicate a good fit.

Although we generally used the overall best set of parameters for statistical tests, we also performed a bootstrapping procedure to make sure that variability among the parameter sets (which could be large in case of poor estimation quality) was also accounted for. For this purpose we generated 10000 samples of individual parameter sets, where one of the 10 final best sets of parameters was randomly assigned for each individual. Then, statistical tests were performed for each of the 10000 samples and the median P-value would reflect the statistical relationship of interest with uncertainty of the parameter estimation included.

### GWAS statistics

GWAS and the replication study were run under the assumption of an additive model. Bonferroni (family-wise error) correction was used to correct for genome-wide multiple testing with significance level of 5%. Golden Helix SNP and Variation Suite 7™ (SVS7, version 7.3.1), Matlab 2008a (The Mathworks Inc., Natick, MA, USA), and PLINK! Software package v1.07 [40] were used for statistical analyses.

### Data analysis of cortical gene expression data

Data are based on the survey of genetic human cortical gene expression [32]. Gene expression studies of 193 samples from the cerebral cortex of neuropathologically normal brains were carried out with the Illumina HumanRefseq-8 Expression BeadChip (Illumina Inc., San Diego, CA, USA). For genome-wide genotyping, the Affymetrix GeneChip Human Mapping 500K Array Set was used. The complete data files were downloaded from http://labs.med.miami.edu/myers/. *BAIAP2* transcript probe was GI_9257196 (NM_017450.1) and expression levels of GI_9257196 were used as a dependent variable. The genetic association analysis was run under the assumption of an additive model.

### The picture task and fMRI

#### Participants

After excluding outliers based on population stratification and age, a total of 451 healthy subjects were used for the study. The subjects were free of any lifetime neurological or psychiatric illness, and did not take any medication at the time of the experiment (except hormonal contraceptives).

#### Procedure

After receiving general information about the study and giving their informed consent, participants were instructed and then trained on the picture task they later performed in the scanner. After training, they were positioned in the scanner. The participants received earplugs and headphones to reduce scanner noise. Their head was fixated in the coil using small cushions, and they were told not to move their heads. Functional MR-images were acquired during the performance of the picture task for approximately 30 min. After finishing the tasks, participants left the scanner and were taken to a different room for free recall of the pictures. Finally, participants filled out questionnaires, gave saliva for genotype analysis and were debriefed. Participants received 25 CHF/h for participation.

#### The picture task

Stimuli consisted of 72 pictures that were selected from the International Affective Picture System (IAPS [41]) as well as from in-house standardized picture sets that allowed us to equate the pictures for visual complexity and content (e.g. human presence). On the basis of normative valence scores (from 1 to 9), pictures were assigned to emotionally negative (2.3±0.6), emotionally neutral (5.0±0.3) and emotionally positive (7.6±0.4) conditions, resulting in 24 pictures for each emotional valence. Participants were not told that they had to remember the pictures for later recall. Participants were instructed to passively view the pictures and subsequently rate them according to emotional valence/arousal (for further details see **Text S1**). 10 minutes after picture presentation, memory performance was tested using a free recall task, which required participants to write down a short description (a few words) of the previously seen pictures. Remembered primacy and recency pictures as well as training pictures were excluded from the analysis. No time limit was set for this task. Two trained investigators independently rated the descriptions for recall success (inter-rater reliability > 99%).

#### Phenotype

As the picture task was used to test the GWAS result from the verbal task (rs8067235 associated with negative modulation of memory), the main phenotype of interest here was the number of negative pictures remembered in the free recall minus the number of neutral pictures remembered. Computational modeling was not applied to this task because of the lack of free recall data at two distinct time points – immediately after encoding and after a delay – that would be needed to provide a sufficient number of different PMs and allow distinguishing learning rates from forgetting/repetition parameters.

#### fMRI contrasts and analyses

To investigate neural correlates of association with the negative modulation of memory strength, the interaction between brain activity during encoding of (negative pictures subsequently remembered vs. forgotten) vs. (neutral pictures subsequently remembered vs. forgotten) was calculated individually using a fixed effects model (first level analysis). Because of using such contrasts, possible artifacts unrelated to underlying neural activity were subtracted. The resulting contrast parameters were then used for genotype-dependent analyses in a random effects model (second level analysis). Specifically, we used a regression model to analyze gene-dose dependent differences in brain activity (with the number of A alleles as covariate). According to previous reports on brain regions involved in successful memory encoding and its emotional modulation [24], [28]–[30], we focused on the MTL memory system, including left and right hippocampi, amygdalae, parahippocampal, and entorhinal cortices. We defined our Region of Interest (ROI) using a 2-step procedure. First we defined an anatomical search mask of the MTL memory system using a study-specific anatomical probabilistic atlas based on FreeSurfer [42] segmentations of individual T1 images (**Text S1**). A 50% probability threshold was applied to each of the analyzed regions of the atlas prior to concatenation. In the second step, we used this search mask on the group level (independent of genotype) to identify voxels that showed a Dm effect (subsequently remembered vs. forgotten) for negative and/or neutral pictures at P<0.001 nominal significance level, as we expected the *BAIAP2* genotype to affect the balance between successful negative and neutral encoding. The combined voxels of negative and neutral Dm defined the final ROI (overall number of voxels in the mask: k = 357). Small volume correction was applied for the mask (family-wise error correction, p<0.05).

## Supporting Information

Figure S1 **Parameter estimation results for the second best model (with fixed parameters σ, s and c).** (**A**) The hill-climbing results of estimating three fixed parameters are shown, with bigger circles and lighter colors indicating better goodness-of-fit; ten best hill-climbing points (biggest orange circles) were selected for evaluating averages of all their possible combinations (as shown in **B**). Circle size and color scale corresponds exactly to that of **Figure 2A**. (**B**) Ten combinations with the best goodness-of-fit are displayed. The best fit was achieved with Gaussian noise σ = 0.7, sigmoidal steepness *s* = 4.133, and repetition-based memory improvement *c* = 1.187. Although averaging combinations led to improvement of goodness-of-fit compared to the best hill climbing point (χ^2^ = 1.543), they remained significantly worse than the goodness-of-fits of combinations from the best model (with forgetting rate γ fixed instead of *c*, **Figure 2B**).
(TIF)

Figure S2 **Significance of association of SNPs in the** ***BAIAP2*** **locus with ε_neg_ in the GWAS sample.** Red dots: Array-based SNPs. Blue dots: Imputed SNPs. The lower panel visualizes the position of known transcripts in the displayed chromosomal region.
(TIF)

Figure S3 **Genomic region harboring** ***BAIAP2*** **(chr17:79008947-79091232, UCSC Genome Browser on Human Feb. 2009 (GRCh37/hg19) Assembly).** Upper panel: Overlaid *H3K27Ac* tracks indicating possible enhancer activity are shown in magenta. Digital DNaseI Hypersensitivity Clusters [31], which are indicative of transcriptional regulatory regions, are shown as bold type black horizontal lines. Lower panel: Magnification of the region harboring rs8067235. This SNP is located within an *H3K27Ac* histone mark and a DNaseI hypersensitivity site.
(TIF)

Figure S4 **Association with** ***BAIAP2*** **cortical expression levels.** The *BAIAP2* SNP rs8070741 is significantly associated with expression levels of *BAIAP2* transcript GI_9257196 in the cortex of 193 non-demented deceased subjects. Black bars indicate mean expression levels of GI_9257196; error bars are s.e.m. Statistics were run under the assumption of an additive genetic model. There were 63 *GG* carriers, 93 *AG* carriers and 37 *AA* carriers.

Table S1 **Results of the model selection procedure.** For each possible choice of 3 parameters being fixed across the population we estimated average goodness-of-fit χ^2^ of individual estimations of the 5 remaining parameters. As performing individual and fixed parameter estimations for each of 56 possible 3-out-of-8 choices was computationally intensive, at this stage fixed parameters were estimated approximately and further refinement performed for the 2 best-fitting models (with fixed parameters {γ, σ, s} and {σ, c, s} – see **Figure 2A** and **Figure S1A**). The analysis revealed that forgetting rates γ and repetition-based memory improvements *c* were strongly related: keeping both of them free led to poor χ^2^ values, whereas the only difference between the two best models was which of γ or *c* was fixed.
(TIF)

Table S2 **Association between** ***BAIAP2*** **rs8067235 genotype and performance measures related to negative modulation of verbal memory.** Significance is calculated based on the additive genetic model. ρ: Spearman's rho.
(PDF)

Text S1 **Supplementary materials, methods and references.**
(PDF)
