# Correction: *BAIAP2* Is Related to Emotional Modulation of Human Memory Strength

**DOI:** 10.1371/journal.pone.0092933

**Published:** 2014-03-17

**Authors:** The PLOS ONE Staff

The Table 3 footnote is missing. This error occurred while the manuscript was being prepared for publication. Please see the corrected Table 3 below.

**Table 3:** Genotype-independent subsequent memory (Dm) analysis for negative and neutral pictures.

Contrast	Region	No. of voxels	L/R	Peak MNI coordinates (x, y, z)	T value	P value
Negative Dm	amygdala	60	R	19, -6, -16	7.94	<10^-6^
		46	L	-22, -6, -16	7.83	<10^-6^
Negative Dm	hippocampus	84	R	19, -11, -16	6.11	<10^-6^
		71	L	-17, -11, -16	5.06	<10^-6^
Negative Dm	parahippocampal cortex	25	R	19, -28, -16	5.71	<10^-6^
		7	L	-28, -30, -20	3.73	0.0001
Negative Dm	entorhinal cortex	10	L	-30, -8, -32	4.95	<10^-6^
		3	R	30, -2, -36	3.84	0.0001
Neutral Dm	parahippocampal cortex	23	L	-17, -41, -12	4.24	10^-5^
		14	R	25, -41, -8	3.67	0.0001
		2	L	-17, -19, -24	3.37	0.0004
Neutral Dm	hippocampus	9	L	-28, -36, -8	4.08	3 ⋅ 10^-5^
		8	L	-17, -17, -24	3.87	0.0001
		2	L	-11, -39, 4	3.37	0.0004

Clusters with voxels at P < 0.001 significance level are shown.
