# Supplementary figures and images for: BAIAP2 Is Related to Emotional Modulation of Human Memory Strength
Source: PLoS One. 2014 Jan 2;9(1):e83707. doi: 10.1371/journal.pone.0083707 (PMC3879265; doi:10.1371/journal.pone.0083707)

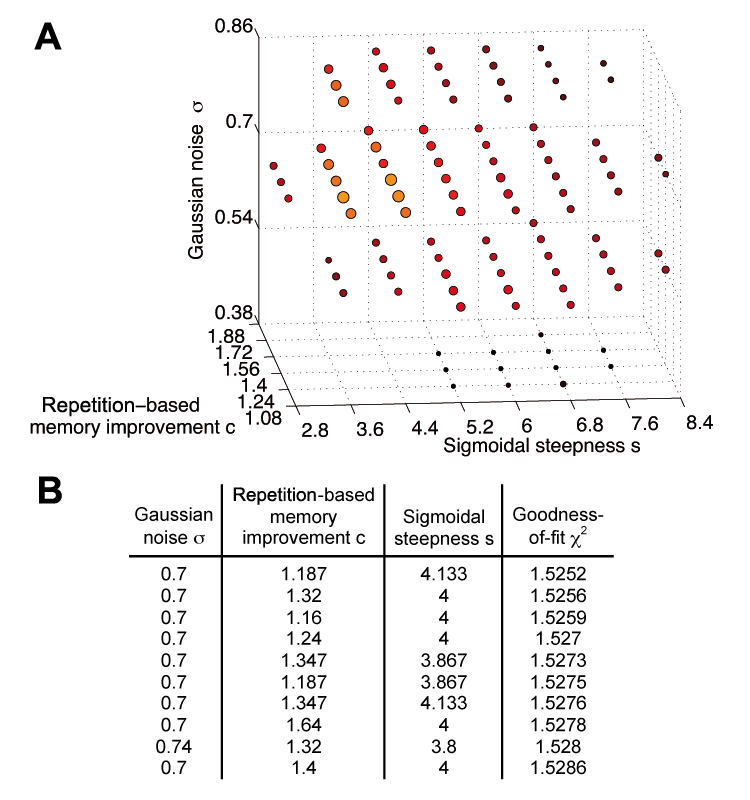

Supplement: Figure S1 — Parameter estimation results for the second best model (with fixed parameters σ, s and c). (A) The hill-climbing results of estimating three fixed parameters are shown, with bigger circles and lighter colors indicating better goodness-of-fit; ten best hill-climbing points (biggest orange circles) were selected for evaluating averages of all their possible combinations (as shown in B). Circle size and color scale corresponds exactly to that of Figure 2A . (B) Ten combinations with the best goodness-of-fit are displayed. The best fit was achieved with Gaussian noise σ = 0.7, sigmoidal steepness s = 4.133, and repetition-based memory improvement c = 1.187. Although averaging combinations led to improvement of goodness-of-fit compared to the best hill climbing point (χ2 = 1.543), they remained significantly worse than the goodness-of-fits of combinations from the best model (with forgetting rate γ fixed instead of c, Figure 2B ). (TIF) [file pone.0083707.s001.tif]

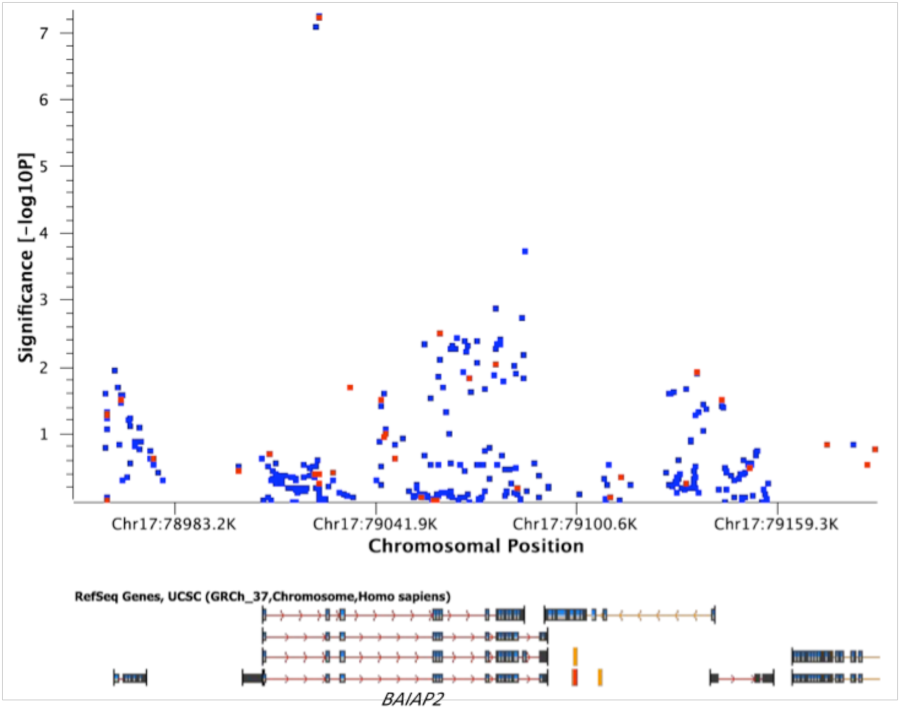

Supplement: Figure S2 — Significance of association of SNPs in the BAIAP2 locus with εneg in the GWAS sample. Red dots: Array-based SNPs. Blue dots: Imputed SNPs. The lower panel visualizes the position of known transcripts in the displayed chromosomal region. (TIF) [file pone.0083707.s002.tif]

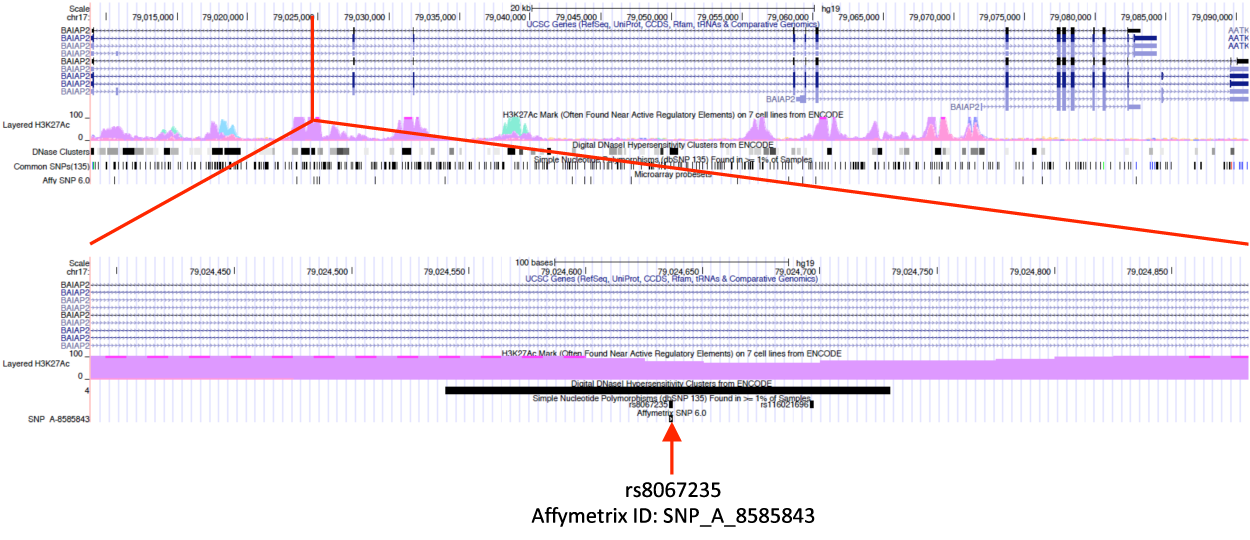

Supplement: Figure S3 — Genomic region harboring BAIAP2 (chr17:79008947-79091232, UCSC Genome Browser on Human Feb. 2009 (GRCh37/hg19) Assembly). Upper panel: Overlaid H3K27Ac tracks indicating possible enhancer activity are shown in magenta. Digital DNaseI Hypersensitivity Clusters [31], which are indicative of transcriptional regulatory regions, are shown as bold type black horizontal lines. Lower panel: Magnification of the region harboring rs8067235. This SNP is located within an H3K27Ac histone mark and a DNaseI hypersensitivity site. (TIF) [file pone.0083707.s003.tif]

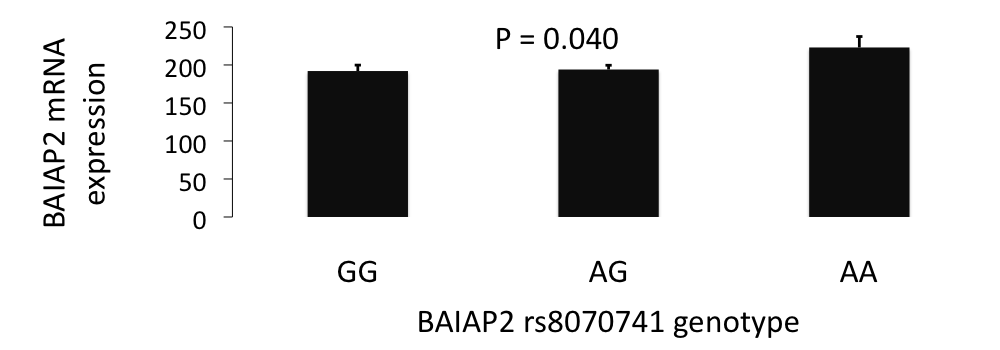

Supplement: Figure S4 — Association with BAIAP2 cortical expression levels. The BAIAP2 SNP rs8070741 is significantly associated with expression levels of BAIAP2 transcript GI_9257196 in the cortex of 193 non-demented deceased subjects. Black bars indicate mean expression levels of GI_9257196; error bars are s.e.m. Statistics were run under the assumption of an additive genetic model. There were 63 GG carriers, 93 AG carriers and 37 AA carriers. [file pone.0083707.s004.tif]
