# Supplementary material for: BAIAP2 Is Related to Emotional Modulation of Human Memory Strength
Source: PLoS One. 2014 Jan 2;9(1):e83707. doi: 10.1371/journal.pone.0083707 (PMC3879265; doi:10.1371/journal.pone.0083707)
Supplement: Table S2 — Association between BAIAP2 rs8067235 genotype and performance measures related to negative modulation of verbal memory. Significance is calculated based on the additive genetic model. ρ: Spearman's rho. (PDF) [file pone.0083707.s006.pdf]

Table S2. Association between *BAIAP2* rs8067235 genotype and performance measures related to negative modulation of verbal memory.

rs8067235 genotype	N	Proportions of negative minus neutral words recalled *immediately*, mean ± s.e.m.	Proportions of negative minus neutral words recalled *after 5 min*, mean ± s.e.m.
AA	137	0.128 ± 0.015	0.080 ± 0.015
AG	532	0.100 ± 0.007	0.025 ± 0.007
GG	570	0.077 ± 0.007	0.001 ± 0.007
		$P = 0.0012$, $\rho = 0.092$	$P = 0.00016$, $\rho = 0.107$
