# Supplementary material for: BAIAP2 Is Related to Emotional Modulation of Human Memory Strength
Source: PLoS One. 2014 Jan 2;9(1):e83707. doi: 10.1371/journal.pone.0083707 (PMC3879265; doi:10.1371/journal.pone.0083707)
Supplement: Text S1 — Supplementary materials, methods and references. (PDF) [file pone.0083707.s007.pdf]

Text S1. Supplementary materials, methods and references.

Genotype imputation

Imputation was performed with IMPUTE v2.2.2, which phases observed genotypes and imputes missing genotypes using a multi-population reference panel [*43, 44*]. All SNPs in the *BAIAP2* region (plus/minus 50 kb) were imputed using the latest available panel data (https://mathgen.stats.ox.ac.uk/impute/data_download_1000G_phase1_integrated.html), derived from the phase1 integrated variant call set of the 1000 Genomes project (release v3 in NCBI build 37/hg19 coordinates, March 2012). Only genotype calls exceeding a probability score of 90% were considered in the subsequent statistical analysis. Additionally all SNPs that failed the following quality controls were removed from the analysis: minor allele frequency > 0.01, SNP call rate > 90%, P-value for Hardy-Weinberg equilibrium > 0.001.

Singleplex SNP genotyping

Singleplex genotyping for rs8067235 was done with Pyrosequencing on a PyroMark™ID System (QIAGEN, Hilden, Germany). Forward primer: 5'-ACA CGC ACA CCA GAA CAC TAG AG-3'; reverse primer (5'-biotinylated): 5'- AGC AGA AGG GGG CAT CAG-3'; sequencing primer: 5'-GCA CAC CAG AAC ACT AGA G-3'.

Array-based SNP genotyping

Blood was drawn by using 2 x 9ml EDTA tubes (Sarstedt, Germany). Saliva was collected with the Oragene® DNA sample collection kit (DNA Genotek Inc., Ontario, Canada). DNA isolation was done according to standard protocols.

Samples were processed as described in the Genome-Wide Human SNP Nsp/Sty 6.0 User Guide (Affymetrix). Briefly, genomic DNA concentration was determined by using a Nano-Drop ND-1000 and adjusted to 50ng/µl in water. 250ng of DNA was digested in parallel with 10 units of Sty I and Nsp I restriction enzymes (New England Biolabs, Beverly, MA) for 2 hours at 37°C. Enzyme specific adaptor oligonucleotides were then ligated onto the digested ends with T4 DNA Ligase for 3 hours at 16°C. After adjustment to 100µl with water, 10µl of the diluted ligation reactions were subjected to PCR. Three PCR reactions of 100µl were performed for Sty digested products and four PCR reactions for Nsp. PCR was performed with Titanium Taq DNA Polymerase (Clontech, Mountain View, CA) in the presence of 4.5 µM PCR primer 002 (Affymetrix), 350 µM each dNTP (Clontech), 1M G-C Melt (Clontech), and 1X Titanium Taq PCR Buffer (Clontech). Cycling parameters were as follows: initial denaturation at 94°C for 3 minutes, amplification at 94°C for 30 seconds, 60°C for 45 seconds and extension at 68°C for 15 seconds repeated a total of 30 times, final extension at 68°C for 7 minutes. Reactions were then verified to migrate at an average size between 200-1100 bps using 2% TBE gel electrophoresis. PCR products were combined and purified with the Filter Bottom Plate

(Seahorse Bioscience, North Billerica, MA) using Agencourt Magnetic Beads (Beckman Coulter, Fullerton, CA). Purified PCR products were quantified on a Zenith 200rt microplate reader (Anthos-Labtec, Cambridge, UK). 4 to 5µg/µl were obtained on average for each sample. From this stage on, the SNP Nsp/Sty 5.0/6.0 Assay Kit (Affymetrix) was used. Around 250 µg of purified PCR products were fragmented using 0.5 units of DNAse I at 37°C for 35 minutes. Fragmentation of the products to an average size less than 180 bps was verified using 4% TBE gel electrophoresis. Following fragmentation, the DNA was end labelled with 105 units of terminal deoxynucleotidyl transferase at 37°C for 4 hours. The labelled DNA was then hybridized onto Genome-Wide Human SNP 6.0 Array at 50°C for 18 hours at 60 rpm. The hybridized array was washed, stained, and scanned according to the manufacturer's (Affymetrix) instructions using Affymetrix GeneChip Command Console (AGCC, version 3.0.1.1214). Generation of SNP calls and Array quality control were performed using the command line programs of the Affymetrix Power Tools package (version: apt-1.12.0). According to the manufacturer's recommendation, Contrast QC was chosen as QC metric, using the default value of greater or equal than 0.4. Mean Call Rate for all samples averaged 98.9%. All samples passing QC criteria were subsequently genotyped using the Birdseed (v2) algorithm.

Detection of outliers based on population stratification and age

Population stratification was assessed by analyzing all genome-wide, array-based autosomal SNPs passing the QC criteria with EIGENSTRAT [*45*]. Principal component analysis (PCA) was applied to each population (Zurich words sample, Basel words sample, and Basel pictures/fMRI sample) to reduce genetic variation to a few dimensions. For PCA, default parameters were used (i.e. definition of ten principal components in five iterations, outlier criterion was six standard deviations). EIGENSTRAT identified a number of individuals deviating from a large genetically homogenous population cluster: 112 in the Zurich sample, 68 in the Basel words sample, and 58 in the Basel pictures/fMRI sample. These outliers were removed from further analyses.

As learning and memory abilities deteriorate with age [*46*], we excluded the outliers (individuals with age further than 2 standard deviations from the mean in each population) from further analyses: 18 in the Zurich words sample, 44 in the Basel words sample, and 24 in the Basel pictures/fMRI sample.

Computation of modeled performance measures ($PM_{1\text{-}8}^{mod}$) based on model parameters (α, β, γ, ε_{pos}, ε_{neg}, s, c, σ)

$$PM_1^{\mathrm{mod}} = 10\int_{\beta}^{\infty} \mathrm{N}\left(m, \alpha\varepsilon_{pos}, \sigma\right) p_{correct}\left(m, s\right) dm$$

The integral evaluates the number of positive words that are correctly recalled immediately. We integrate m from β, as this is the smallest memory strength necessary to attempt the recall. After encoding, the m values for positive words are normally distributed with mean $\alpha\varepsilon_{pos}$ and standard deviation σ.

$$PM_2^{\mathrm{mod}} = 10\int_{\beta}^{\infty} \mathrm{N}(m,\alpha\varepsilon_{neg},\sigma)\, p_{correct}(m,s)\,dm$$

Same as PM_1^{mod}, just we consider negative words (for which the mean memory strength after encoding is $\alpha\varepsilon_{neg}$ instead of $\alpha\varepsilon_{pos}$).

$$PM_3^{\mathrm{mod}} = 10\int_{\beta}^{\infty} \mathrm{N}(m,\alpha,\sigma)\, p_{correct}(m,s)\,dm$$

Same as PM_1^{mod} and PM_2^{mod}, just we consider neutral words (for which the mean memory strength after encoding is α).

$$PM_4^{\mathrm{mod}} = 10\int_{\beta}^{\infty} \left[\mathrm{N}(m,\alpha\varepsilon_{pos},\sigma) + \mathrm{N}(m,\alpha\varepsilon_{neg},\sigma) + \mathrm{N}(m,\alpha,\sigma)\right]\left(1 - p_{correct}(m,s)\right)dm$$

Similar to PM_1^{mod}, PM_2^{mod}, PM_3^{mod}, just all groups (positive, negative, neutral) are added up, because here we consider mistakes – words that were **not** recalled correctly in the immediate recall (nevertheless attempted to recall). Therefore in the last component of the integral we use $1\text{–}p_{correct}$ instead of $p_{correct}$ used in PM_1^{mod}, PM_2^{mod}, PM_3^{mod}.

$$PM_5^{\mathrm{mod}} = 10\int_{\max(\beta,\beta/c\gamma)}^{\infty} \mathrm{N}(m,\alpha\varepsilon_{pos},\sigma)\, p_{correct}(m,s)\, p_{correct}(mc\gamma,s)\,dm +$$
$$+10\int_{\beta/\gamma}^{\infty} \mathrm{N}(m,\alpha\varepsilon_{pos},\sigma)\left(1 - p_{correct}(m,s)\right) p_{correct}(m\gamma,s)\,dm$$

The first integral represents positive words that were recalled correctly in the immediate recall (thus got multiplied by both repetition-based memory improvement *c* and forgetting factor γ) **and** in the 5 min recall. For this to happen, memory strength m must be higher than β both in the immediate recall and after multiplication by $c\gamma$ (therefore we integrate *m* from $max(\beta, \beta/c\gamma)$). The second integral represents positive words that were **not** recalled correctly immediately (thus got multiplied only by forgetting factor γ), but were recalled correctly after 5 min. For the latter to happen, memory strength during the 5 min recall $m\gamma$ must be higher than β (therefore we integrate *m* from β/γ).

$$PM_6^{\mathrm{mod}} = 10\int_{\max(\beta,\beta/c\gamma)}^{\infty} \mathrm{N}(m,\alpha\varepsilon_{neg},\sigma)\, p_{correct}(m,s)\, p_{correct}(mc\gamma,s)\,dm +$$
$$+10\int_{\beta/\gamma}^{\infty} \mathrm{N}(m,\alpha\varepsilon_{neg},\sigma)\left(1 - p_{correct}(m,s)\right) p_{correct}(m\gamma,s)\,dm$$

Same as PM_5^{mod}, just we consider negative words.

$$PM_7^{\text{mod}} = 10 \int_{\max(\beta,\beta/c\gamma)}^{\infty} \mathrm{N}(m,\alpha,\sigma) p_{correct}(m,s) p_{correct}(mc\gamma,s) dm +$$

$$+10 \int_{\beta/\gamma}^{\infty} \mathrm{N}(m,\alpha,\sigma)\left(1 - p_{correct}(m,s)\right) p_{correct}(m\gamma,s) dm$$

Same as PM_5^{mod} and PM_6^{mod}, just we consider neutral words.

$$PM_8^{\text{mod}} = 10 \int_{\max(\beta,\beta/c\gamma)}^{\infty} \left(\mathrm{N}(m,\alpha\varepsilon_{pos},\sigma) + \mathrm{N}(m,\alpha\varepsilon_{neg},\sigma) + \mathrm{N}(m,\alpha,\sigma)\right) p_{correct}(m,\beta)\left(1 - p_{correct}(mc\gamma,\beta)\right) dm$$

$$+10 \int_{\beta/\gamma}^{\infty} \left(\mathrm{N}(m,\alpha\varepsilon_{pos},\sigma) + \mathrm{N}(m,\alpha\varepsilon_{neg},\sigma) + \mathrm{N}(m,\alpha,\sigma)\right)\left(1 - p_{correct}(m,\beta)\right)\left(1 - p_{correct}(m\gamma,\beta)\right) dm$$

Similar to PM_5^{mod}, PM_6^{mod}, PM_7^{mod}, just all groups (positive, negative, neutral) are added up, because here we consider mistakes – words that were **not** recalled correctly in the 5 min recall (nevertheless attempted to recall). Therefore in the last component of the integrals we use $1–p_{correct}$ instead of $p_{correct}$ used in PM_5^{mod}, PM_6^{mod}, PM_7^{mod}.

Notes: N(m, λ, σ) is a normal probability density function with variable m (denoting modeled memory strength), mean λ, and standard deviation σ;

$p_{correct}$(m, s) is probability to recall a word with memory strength m correctly (which depends on sigmoidal steepness s);

10 in front of the integrals represents 10 words in each (positive, negative, neutral) group.

Further details of the picture task

Participants were trained on the picture task before being positioned in the scanner. Training consisted of presentation and rating of five pictures including scenes and scrambled pictures, which were not used during scanning. Four additional pictures showing neutral objects were used to control for primacy and recency effects in memory. Two of these pictures were presented in the beginning and two at the end of the picture task. They were not included in the analysis. In addition, 24 scrambled pictures were used. The background of the scrambled pictures contained the color information of all pictures used in the experiment (except primacy and recency pictures), overlaid with a crystal and distortion filter (Adobe Photoshop CS3). In the foreground, a mostly transparent geometrical object (rectangle or ellipse of different sizes and orientations) was shown. Pictures were presented in the scanner using MR-compatible LCD goggles (Visuastim XGA, Resonance Technology, Los Angeles, CA). Eye correction was used when necessary.

The pictures were presented for 2.5 seconds in a quasi-randomized order so that at maximum four pictures of the same category occurred consecutively. A fixation-cross appeared on the screen for 500 ms before each picture presentation. Trials were separated by a variable inter-trial period of 9-12 sec (jitter) that was equally distributed for each stimulus category. During the inter-trial period, participants subjectively rated the picture

showing scenes according to valence (negative, neutral, positive) and arousal (large, medium, small) on a three-point scale (Self Assessment Manikin, SAM) by pressing a button with a finger of their dominant hand. For scrambled pictures, participants rated form (vertical, symmetric or horizontal) and size (large, medium, small) of the geometrical object in the foreground.

Construction of a population-average anatomical probabilistic atlas

Automatic segmentation of the subjects' T1-weighted images was used to build a population-average probabilistic anatomical atlas. More precisely, each participant's T1-weighted image was first automatically segmented into cortical and subcortical structures using FreeSurfer (version 4.5, http://surfer.nmr.mgh.harvard.edu [*42*]). Labeling of the cortical gyri was based on the Desikan-Killiany Atlas [*47*], yielding 35 regions per hemisphere.

The segmented T1 image was then normalized to the study-specific anatomical template space using the subject's previously computed warp field, and affine-registered to the MNI space. Nearest-neighbor interpolation was applied, in order to preserve labeling of the different structures. The normalized segmentations were finally averaged across subjects, in order to create a population-average probabilistic atlas. Each voxel of the template could consequently be assigned a probability of belonging to a given anatomical structure, based on the individual information from all subjects.

fMRI data acquisition and pre-processing

Measurements were performed on a Siemens Magnetom Verio 3 T whole-body MR unit equipped with a twelve-channel head coil. Functional time series were acquired with a single-shot echo-planar sequence using parallel imaging (GRAPPA). We used the following acquisition parameters: TE (echo time) = 35 ms, FOV (field of view) = 22 cm, acquisition matrix = 80 × 80, interpolated to 128 × 128, voxel size: 2.75 × 2.75 × 4 mm^3, GRAPPA acceleration factor R = 2.0. Using a midsaggital scout image, 32 contiguous axial slices placed along the anterior–posterior commissure (AC–PC) plane covering the entire brain with a TR = 3000 ms (α = 82°) were acquired using an ascending interleaved sequence. A high-resolution T1-weighted anatomical image was acquired using a magnetization prepared gradient echo sequence (MPRAGE, TR=2000 ms; TE=3.37 ms; TI=1000 ms; flip angle=8; 176 slices; FOV= 256 mm).

Pre-processing and data analysis was performed using SPM8 (Statistical Parametric Mapping, Wellcome Trust Centre for Neuroimaging, London, UK; http://www.fil.ion.ucl.ac.uk/spm/) implemented in Matlab R2011b (The Mathworks Inc., Natick, MA, USA). Volumes were slice-time corrected to the first slice and realigned using the 'register to mean' option. A mean image was generated from the realigned series and co-registered to the structural image. This ensured that functional and structural images were spatially aligned.

The functional images and the structural images were spatially normalized by applying DARTEL, which leads to an improved registration between subjects. Normalization incorporated the following steps: 1. Structural images of each subject were segmented using the "New Segment" procedure in SPM8. 2. The resulting gray and white matter images were used to derive a study-specific group template. The template was computed from a large population of 612 subjects that included the 435 subjects from this study. 3. An affine transformation was applied to map the group template to MNI space. 4. Subject-to-template and template-to-MNI transformations were combined to map the functional images to MNI space. The functional images were smoothed with an isotropic 8 mm full width at half maximum (FWHM) Gaussian filter.

Intrinsic autocorrelations were accounted for by AR(1) and low-frequency drifts were removed via high-pass filter (time constant 128 s). For each subject, evoked hemodynamic responses to event-types were modelled with a delta function corresponding to presentation of each stimulus category (negative, positive, neutral and scrambled pictures, respectively) convolved with a canonical hemodynamic response function within the context of a general linear model (GLM). Button presses and rating scale presentation during valence and arousal ratings were modelled separately. In addition, six movement parameters from spatial realigning were included as regressors of no interest. Pictures accounting for possible primacy and recency effects were excluded from statistical analysis.

fMRI group statistics

EPI sequences suffer from signal loss in the presence of magnetic field inhomogeneities that can occur close to air-tissue boundaries. The normalization procedure applied in DARTEL accurately transforms both voxels with signal and voxels with signal loss to MNI space. In SPM8, signal loss at a MNI coordinate in a functional image of only one subject leads to the exclusion of the voxel at this coordinate from the group level analysis. Therefore, the probability of a voxel being excluded increases with sample size. GLM Flex circumvents this problem by allowing a variable number of subjects at each voxel, (Martinos Center & Mass General Hospital, Charlestown, MA, USA; http://nmr.mgh.harvard.edu/harvardagingbrain/People/AaronSchultz/Aarons_Scripts.html). The minimum number of subjects per voxel was set to be 150.

References

43. Howie BN, Donnelly P, Marchini J (2009) A flexible and accurate genotype imputation method for the next generation of genome-wide association studies. *PLoS Genet* **5**:e1000529
44. Howie B, Marchini J, Stephens M (2011) Genotype imputation with thousands of genomes. *G3 (Bethesda)* **1**:457–470.
45. Price AL, *et al*. (2006) Principal components analysis corrects for stratification in genome-wide association. *Nature Genet* **38**:904–909.
46. Nilsson LG (2003) Memory function in normal aging. *Acta Neurol Scand Suppl* **179**:7–13.
47. Desikan RS, *et al*. (2006) An automated labeling system for subdividing the human cerebral cortex on MRI scans into gyral based regions of interest. *NeuroImage* **31**:968–980.
